# Nerve Ultrasound Distinguishes Non-Inflammatory Axonal Polyneuropathy From Inflammatory Polyneuropathy With Secondary Axonal Damage

**DOI:** 10.3389/fneur.2021.809359

**Published:** 2022-01-28

**Authors:** Jil Brünger, Jeremias Motte, Thomas Grüter, Hannah Mork, Yesim Bulut, Anne Carolus, Diamantis Athanasopoulos, Min-Suk Yoon, Ralf Gold, Kalliopi Pitarokoili, Anna Lena Fisse

**Affiliations:** ^1^Department of Neurology, St. Josef Hospital, Ruhr-University Bochum, Bochum, Germany; ^2^Immunmediated Neuropathies Biobank (INHIBIT), Ruhr University Bochum, Bochum, Germany; ^3^Clinic for Neurosurgery, University Medical Center Knappschaftskrankenhaus Bochum, Bochum, Germany; ^4^Department of Neurology, Evangelisches Krankenhaus Hattingen, Hattingen, Germany

**Keywords:** chronic inflammatory demyelinating polyneuropathy, axonal damage, Bochum ultrasound score, high-resolution ultrasonography, inflammatory neuropathy

## Abstract

**Introduction:**

Chronic inflammatory demyelinating polyneuropathy (CIDP) may have a similar clinical and electrophysiological presentation to non-inflammatory axonal polyneuropathies (NIAPs) when secondary axonal damage occurs. We aimed to investigate if nerve ultrasound can help to differentiate CIDP with additional secondary axonal damage from NIAP.

**Methods:**

In a retrospective analysis, the cross-sectional area (CSA) of the peripheral nerves measured by ultrasound at six suitable nerve sites was compared in 95 patients with CIDP and 82 patients with NIAP. We developed the adjusted Bochum ultrasound score (aBUS) ranging from 0 to 6 resulting from the number of sites with enlarged CSA (median, ulnar, radial, and sural nerve).

**Results:**

The mean CSA of patients with CIDP was enlarged at all six nerve sites compared with the mean CSA of patients with NIAP. A total of 21 patients with CIDP did not meet 2010 electrophysiological diagnostic criteria (European Academy of Neurology/Peripheral Nerve Society Guideline, EFNS/PNS criteria) for CIDP at examination timepoint but only in further follow-up, while 25 patients with NIAP fulfilled electrophysiological EFNS/PNS criteria for CIDP as “possible” or “probable” CIDP. To increase diagnostic power, we included aBUS measured by ultrasound in patients classified as “possible” or “probable” resulting in an improved specificity of 94% and a sensitivity of 59%, compared to a specificity of the EFNS/PNS criteria alone of 60% and sensitivity of 78%.

**Conclusion:**

Using nerve ultrasound and the aBUS as a complementary method to distinguish CIDP from NIAP in case of secondary axonal damage can facilitate the diagnosis of CIDP.

## Introduction

Chronic inflammatory neuropathies may have a similar clinical appearance as non-inflammatory axonal polyneuropathies (NIAPs). Typical chronic inflammatory demyelinating polyneuropathy (tCIDP) and distal acquired demyelinating symmetric neuropathy (DADS), which is considered as an atypical CIDP variant (1), can resemble clinical symptoms of diabetic or toxic polyneuropathy. In CIDP with additional secondary axonal damage distinction from NIAP can be challenging, especially in the late stages of the disease even with the use of thorough nerve conduction studies (NCS) when there is complete denervation. On the one hand, the electrophysiologic features of demyelination, which are necessary to diagnose CIDP according to the European Federation of Neurological Societies/Peripheral Nerve Society (EFNS/PNS) criteria (2), might not be fulfilled in patients with axonal damage. On the other hand, some patients with NIAP also meet the electrophysiological EFNS/PNS criteria for possible or even probable CIDP. In clinical practice, the difficult differentiation of CIDP, especially DADS and other atypical variants, with axonal damage and NIAP is a common problem (3), which results in trial and error using immunotherapy. Patients may be treated for months even though the diagnosis of inflammatory polyneuropathy is not confirmed and, vice versa, patients with axonal polyneuropathies of inflammatory origin are not recognized and are not treated in time (4).

In recent years, nerve ultrasound was shown to be useful as an additional tool to NCS to differentiate polyneuropathies. It helps to distinguish between acute and chronic inflammatory polyneuropathies (5) and hereditary from inflammatory polyneuropathies (6). Also, the usefulness of nerve ultrasound in the distinction of NIAP from demyelinating inflammatory polyneuropathies was described in few studies with a small number of patients (6–8). However, it has not yet been shown, if nerve ultrasound helps to distinguish CIDP with (secondary) axonal damage from NIAP, which is a crucial problem in daily clinical practice.

The aim of this study was to investigate if nerve ultrasound can help to differentiate CIDP with additional secondary axonal damage from NIAP. We aimed to find out at which step of the diagnostic pathway high-resolution nerve ultrasound can increase diagnostic discriminatory power.

## Methods

### Patients

A total of 67 patients with tCIDP and 28 patients with DADS treated in St. Josef- Hospital, University Hospital Bochum, Germany between September 2016 and February 2020 were included. Patients with CIDP were diagnosed according to EFNS/PNS criteria (2), at the timepoint of examination, or in the course of the disease. For diagnosis of DADS as an atypical CIDP variant, criteria by Doneddu et al. (9) were used additionally. All patients with CIDP and DADS had additional axonal damage.

The control group of NIAP consisted of 82 patients in total: 40 patients had diabetic polyneuropathy, 12 patients had Oxaliplatin induced polyneuropathy, and 30 patients had idiopathic polyneuropathy associated with restless legs syndrome. Ethical approval was given by the ethics committee of the Ruhr University (Bochum Immunmediated Neuropathies Biobank INHIBIT vote-no. 18-6534-BR, registered DRKS00024494, and votes-no. 18-6407, 4856, and 4905-14), and all patients provided written informed consent. Nerve ultrasound examinations and NCS, which proved axonal damage, were retrospectively evaluated.

Considering all patients, the median time span between NCS and ultrasound examination was 1 day (interquartile range 4 days).

### Ultrasound Examination

All ultrasound studies were performed with an Affiniti 70® (Philips, Hamburg, Germany) or an Aplio® XG ultrasound system (Toshiba Medicals, Tochigi, Japan) by investigators with high neuromuscular ultrasound experience. Settings (e.g., contrast) excluding depth and focus were kept constant during all examinations. Ultrasound examination was performed as previously described in Kerasnoudis et al. (10) and Pitarokoili et al. (11): For the superficial nerves of the lower extremities (sural nerve), an 18-MHz linear array transducer was used. For the deeper nerves, a 12-MHz linear array transducer might have been used. Orientation of the transducer was always kept perpendicular to the nerves to avert anisotropy. To avoid artificial nerve deformity, extremities were kept in the neutral position, and no additional force was applied other than the weight of the transducer. CSA was measured at the inner border of the thin hyperechoic epineural rim by the continuous tracing technique.

Six nerve sites from the whole examination protocol (5) were chosen based on experience regarding feasibility and reliability of CSA measurement in these sites: median nerve at forearm (10 cm proximal to flexor retinaculum) and upper arm (midpoint between medial epicondyle and axillary fossa), ulnar nerve at forearm (10 cm proximal to the Guyon canal) and upper arm (midpoint between medial epicondyle and axillary fossa), radial nerve at upper arm (at spiral groove) and sural nerve at the calf (between the medial and the lateral heads of the gastrocnemius muscle) on both sides of the body. Out of these six nerve sites, we developed the adjusted Bochum ultrasound score (aBUS) with values from 0 to 6. The final score value results from the number of sites with significantly enlarged CSA, whereby we refer to the standard values published by Kerasnoudis et al. (12). If the same measuring point was enlarged on both sides of the body, only 1 point was awarded (Table 1). In comparison to BUS (5), which includes CSA of ulnar nerve at Guyon loge and upper arm, radial nerve, and sural nerve, the aBUS includes different nerve sites.

**Table 1:** Overview of the anatomic sites and scoring system of adjusted Bochum ultrasound score.

**Anatomic sites**	**Points**
CSA of the median nerve in the forearm	1
CSA of the median nerve in the upper arm	1
CSA of the ulnar nerve in the forearm	1
CSA of the ulnar nerve in the upper arm	1
CSA of the radial nerve in spiral groove	1
CSA of the sural nerve between the gastrocnemius muscle	1
**Sum score**	**6**

*CSA, cross-sectional area*.

### Nerve Conduction Studies

All patients went through an electrophysiological examination performed by two investigators with significant experience (JB and KP). A Medtronic four channel electroneurography device (Medtronic, Meerbusch, Germany) and a Natus® Dantec™ Keypoint® Focus or G4 EMG Device (Natusc® Europe GmbH, Planegg, Germany) were used (YB and ALF). The examination protocol for tCIDP and DADS consisted of studies of median, ulnar, radial, fibular, and tibial nerve and sensory studies of the median, ulnar, and sural nerve, which were all performed bilaterally. We used the reference values published by Stöhr and Pfister (13). Measured parameters were distal motor latency (DML), conduction velocity, *f*-wave latency, the amplitude of compound motor action potential (CMAP), and amplitude of sensory nerve action potential (SNAP). The SNAP was calculated after averaging at least 10 responses. Needle electromyography was no part of this study.

Because of the retrospective design of the NIAP control group, these patients did not undergo the full NCS study protocol. However, all of them received NCS during the clinical routine.

We stratified the grade of axonal damage according to the CMAP and SNAP amplitude of the NCS performed at the timepoint of the nerve ultrasound in relation to the reference values on amplitudes of our electrophysiology laboratory. Grading was performed according to the following definition:

- Slight axonal damage: 71 to <100% of the lower limit of the reference amplitude.
- Moderate axonal damage: 41–70% of the lower limit of the reference amplitude.
- Severe axonal damage: 11–40% of the lower limit of the reference amplitude.
- No CMAP or SNAP amplitude detectable: <10% of the lower limit of the amplitude of the reference values.

### Statistical Analysis

The CSA enlargement of the six studied sites was compared between patients with CIDP and NIAP using the Mann–Whitney test for nonparametric variables. The statistically significant threshold was set at a *p*-value <0.05. The Benjamini and Hochberg method was used to control the false discovery rate. Absolute data are presented as mean ± SD.

Statistical analysis was performed using the IBM® SPSS Statistics (version 26.0.0.0; IBM Corp., Armonk, NY, USA) and GraphPad Prism (La Jolla, CA, USA). After the development of the aBUS, specificity, sensitivity, and positive and negative predictive values (PPV and NPV) were calculated for different cut-off point values of the aBUS. Finally, we developed a diagnostic pathway, which includes nerve ultrasound additionally to NCS to differentiate CIDP from NIAP.

## Results

Baseline data, disease characteristics, grade of axonal damage, and diagnostic certainty according to EFNS/PNS criteria are given in Table 2.

**Table 2:** Demographics, disease characteristics, grade of axonal damage, and European Federation of Neurological Societies/Peripheral Nerve Society (EFNS/PNS) criteria of all subgroups.

	**Typical CIDP**		**DADS**		**Diabetic**		**Oxaliplatin induced**		**Idiopathic neuropathy**	
	**(*****n*** **= 67)**		**(*****n*** **= 28)**		**neuropathy**		**induced**		**associated with restless**	
					**neuropathy (*****n*** **= 40)**		**legs syndrome (*****n*** **= 12)**		**(*****n*** **= 30)**	
Gender, male/female	54/22		26/2		19/21		7/5		10/20	
Age at examination, mean, SD	59	±13	60	±11	68	±11	65	±6	64	±18
Disease duration from diagnosis until examination (months), median, range	31	0–295	21	0–118	128	3–366	1	0–5	89	0–397
INCAT-ODSS at time of examination, median, range	2	0–9	2	0–6	n.a.	n.a.	n.a.	n.a.	n.a.	n.a.
Grade of axonal damage ^*^, *n*, %										
Slight	10	15%	2	7%	7	18%	5	42%	10	33%
Moderate	34	51%	19	68%	19	48%	6	50%	12	40%
Severe	18	27%	7	25%	7	18%	1	8%	6	20%
No amplitudes detectable	5	8%	0	0%	7	18%	0	0%	1	3%
EFNS/PNS criteria at timepoint of ultrasound examination, *n*, %										
Not fulfilled	17	25%	4	14%	29	73%	9	75%	19	63%
Possible	16	24%	7	25%	10	25%	2	17%	11	37%
Probable	10	15%	7	25%	1	3%	1	8%	0	0%
Definite	24	36%	10	36%	0	0%	0	0%	0	0%

*INCAT-ODSS, inflammatory neuropathy cause and treatment overall disability score*.

* *Grading of axonal damage*.

*Slight axonal damage: 71 to <100 % of the lower limit of the reference amplitude*.

*Moderate axonal damage: 41–70 % of the lower limit of the reference amplitude*.

*Severe axonal damage: 11–40 % of the lower limit of the reference amplitude*.

*No CMAP or SNAP amplitude detectable: <10 % of the lower limit of the amplitude of the reference values*.

**Figure 1:**
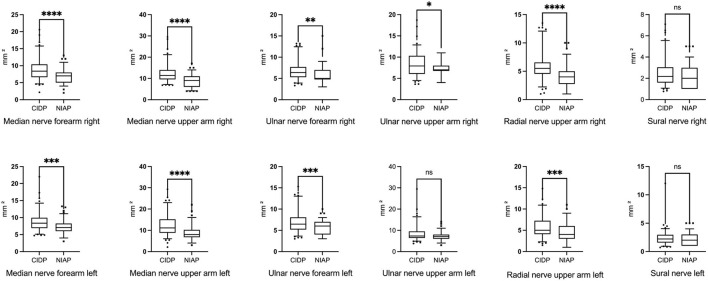
Boxplots of CSA values of all nerve sites showing larger nerve sizes of all examined nerve sites in patients with CIDP compared to patients with NIAP. Differences were statistically significant except in the sural nerve and left ulnar nerve in the upper arm. Corresponding CSA and SD are given in Table 3. *p < 0.05; **p < 0.01, ***p < 0.001, ****p < 0.0001, ns, not significant (Mann–Whitney test). CSA, cross-sectional area; CIDP, chronic inflammatory demyelinating polyneuropathy; NIAP, non-inflammatory axonal polyneuropathy.

Mean CSA of patients with CIDP was enlarged at all six nerve sites compared with mean CSA of patients with NIAP, but the enlargement of the sural nerve and the left ulnar nerve on the upper arm was not statistically significant. Mean absolute CSA values, SD, *p*-values, and matching boxplots are shown in Table 3 and Figure 1.

**Table 3:** Absolute CSA (mm^2^), mean (SD).

	**CIDP**	**NIAP**	***p*-value**
	***n* = 95**	***n* = 82**	
Median nerve forearm, right side	8.9 (3.3)	7.0 (2.2)	<**0.0001**
Median nerve forearm, left side	8.9 (2.9)	7.4 (2.0)	**0.0003**
Median nerve upper arm, right side	12.3 (4.2)	8.8 (3.0)	<**0.0001**
Median nerve upper arm, left side	12.5 (5.0)	8.9 (3.5)	<**0.0001**
Ulnar nerve forearm, right side	6.8 (2.3)	5.8 (2.1)	**0.0012**
Ulnar nerve forearm, left side	7.0 (2.6)	5.6 (1.6)	**0.0002**
Ulnar nerve upper arm, right side	8.3 (2.8)	7.2 (1.7)	**0.0202**
Ulnar nerve upper arm, left side	8.6 (3.8)	7.3 (2.0)	0.0831
Radial nerve, right side	5.9 (2.6)	4.1 (2.2)	<**0.0001**
Radial nerve, left side	5.8 (2.5)	4.4 (2.3)	**0.0001**
Sural nerve, right side	2.5 (1.3)	2.2 (1.0)	0.3767
Sural nerve, left side	2.4 (1.4)	2.2 (1.1)	0.3348

*CSA, cross-sectional area; CIDP, chronic inflammatory demyelinating polyneuropathy, NIAP, non-inflammatory axonal polyneuropathy. Statistically significant p-values are given in bold*.

The aBUS calculated from the enlargement of these sites had a specificity of 83% and sensitivity of 53% when at least 2 points were obtained, a specificity of 95% and a sensitivity of 36% when at least 3 points were obtained, and a specificity of 99% and a sensitivity of 21% when at least 4 points were obtained (Table 4). As a compromise of specificity and sensitivity, we suggest using a cut-off of ≥2 points in the aBUS to diagnose a CIDP.

**Table 4:** Specificity, sensitivity, PPV, and NPV of aBUS for different cut-off values.

	**Specificity %**	**Sensitivity %**	**PPV %**	**NPV %**
6/6 points	98.8	3.8	80.0	44.6
3 5/6 points	98.8	10.4	91.7	46.3
3 4/6 points	98.8	20.8	95.7	49.4
3 3/6 points	95.2	34.0	90.0	53.0
3 2/6 points	83.1	52.8	80.0	58.0
3 1/6 points	47.0	75.5	64.5	60.0

*aBUS, adjusted Bochum ultrasound score; NPV, negative predictive value; PPV, positive predictive value*.

To illustrate the benefits of nerve ultrasound in differentiation of CIDP and NIAP, we calculated specificity and sensitivity of electrophysiological EFNS (2) values with and without additional nerve ultrasound (Table 5). According to electrophysiological EFNS/PNS criteria (2), 34, 17, and 23 patients with CIDP had a definite, probable, and possible CIDP diagnosis, respectively. In 21 cases, the criteria could not be confirmed at the timepoint of the study, but before or later in course of the disease. A total of 57 patients with NIAP did not meet the criteria, but 23 scored “possible” and 2 “probable” CIDP in electrophysiological EFNS criteria, although only a short NCS protocol was applied, even though these patients had other diagnoses considering clinical criteria. This results in a specificity of 70% and a sensitivity of 78% for the use of electrophysiological EFNS/PNS criteria (2) without nerve ultrasound. A total of 9 of 21 CIDP and 9 of 57 patients with NIAP not meeting the EFNS/PNS electrodiagnostic criteria fulfilled the aBUS ≥2.

**Table 5:** Sensitivity, specificity, PPV, and NPV for differentiation of chronic inflammatory neuropathies from NIAP using electrophysiological EFNS/PNS criteria (2) only vs. using aBUS additionally in patients with possible and probable CIDP diagnosis according to electrophysiological EFNS/PNS criteria (2).

	**Specificity %**	**Sensitivity %**	**PPV %**	**NPV %**
Electrophysiological EFNS/PNS	69.5 (58.4–79.2)	77.9 (68.2–85.8)	74.8 (67.7–80.7)	73.1 (64.5–80.3)
Additional aBUS	94.0 (86.3–98.0)	59.0 (48.4–68.9)	91.8 (82.5–96.4)	66.4 (60.7–71.7)
Additional aBUS tCIDP only	93.9 (86.3–98.0)	55.2 (42.6–67.4)	88.1 (75.5–94.7)	72.0 (66.2–77.1)
Additional aBUS DADS only	93.9 (86.3–98.0)	67.9 (47.7–84.1)	79.2 (61.0–90.2)	89.5 (83.3–93.6)

*aBUS, adjusted Bochum ultrasound score; CIDP, chronic inflammatory demyelinating polyneuropathy; tCIDP, typical CIDP; EFNS/PNS, European Federation of Neurological Societies/Peripheral Nerve Society; NIAP, non-inflammatory axonal polyneuropathy; NPV, negative predictive value; PPV, positive predictive value*.

**Figure 2:**
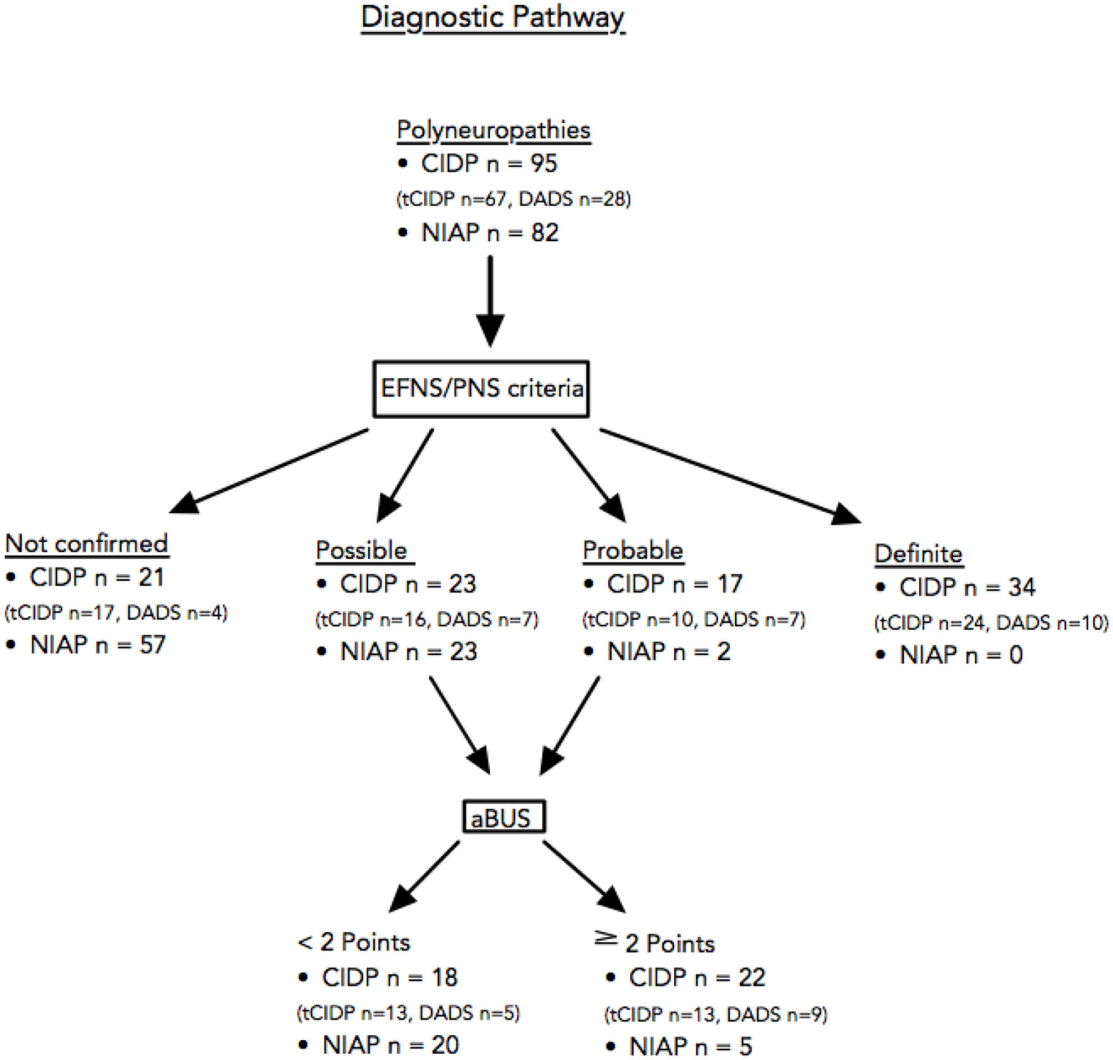
Diagnostic pathway using ultrasound in patients with possible and probable CIDP diagnosis according to electrophysiological EFNS/PNS criteria (2). CIDP, chronic inflammatory demyelinating polyneuropathy; EFNS/PNS, European Federation of Neurological Societies/Peripheral Nerve Society.

To increase diagnostic discriminatory power, we developed the following diagnostic pathway. In all patients classified as “possible” or “probable,” additional nerve ultrasound examination with the calculation of aBUS was performed. Then, for all patients, a specificity of 94% and a sensitivity of 59% was reached (Table 5). In terms of subgroup analysis (Table 5), using the diagnostic pathway only for patients with tCIDP, specificity is 94% and sensitivity is 55%, whereas for patients with DADS, specificity is 94% and sensitivity is 68%. The entire pathway using ultrasound in patients with possible and probable CIDP diagnosis according to electrophysiological EFNS/PNS criteria (2) is shown in Figure 2.

Grade of axonal damage did neither correlate to diagnostic certainty using EFNS/PNS criteria nor to aBUS.

## Discussion

This study shows for the first time that nerve ultrasound is a helpful tool to differentiate between patients with CIDP with secondary axonal damage and NIAP using the newly developed aBUS. The most important finding is that nerve ultrasound is helpful in the diagnosis of chronic inflammatory neuropathies in cases in which electrophysiological EFNS/PNS criteria (2) result in only “possible” or “probable” diagnosis, which for example occurs in the course of the disease due to secondary axonal damage. Diagnostic delay is a common problem in CIDP, so this question is highly relevant for daily clinical practice. Another important finding is that nerve ultrasound is also helpful as a diagnostic tool in the atypical CIDP variant DADS, which cannot be distinguished from NIAP by clinical characteristics.

In conclusion from our findings, we developed a diagnostic pathway, suggesting the use of nerve ultrasound to differentiate between patients with CIDP with secondary axonal damage and NIAP if electrophysiological EFNS/PNS criteria (2) result in diagnostic insecurities.

### Diagnostic Dilemma

There is evidence that diagnosis of CIDP may be difficult in case of additional axonal and nerve ultrasound is particularly helpful to assess the progress of the disease (11, 14). As expected from clinical experience, we showed that nerve ultrasound with measurement of the CSA could be a helpful tool to characterize cases, which cannot be clearly diagnosed regarding EFNS/PNS criteria. This finding is in line with studies, which showed that between NCS and nerve ultrasound there is no significant correlation since each of them evaluates different aspects, namely NCS show functional and nerve ultrasound morphological impairment of the peripheral nerve (15). Additionally, Herraets et al. (8) showed that the use of nerve ultrasound to detect treatment-responsive patients compared to NCS alone had an additional yield of approximately 25%. This underlines the usefulness of nerve ultrasound in detecting patients with inflammatory neuropathies with no characteristic NCS abnormalities found in previous studies (7, 16).

Merola et al. (17) demonstrated that higher CSA values were observed in nerves with predominantly demyelinating features. They speculated that different disease phases might be associated with different ultrasound patterns, an initial/intermediate phase of inflammation, and myelin damage characterized by increased CSA, and a late phase of several axonal degenerations characterized by reduced CSA (17). Based on our results, we can conclude that patients with CIDP despite secondary axonal damage show a greater nerve enlargement than non-inflammatory axonal neuropathies, probably because inflammation still occurs. Enlarged nerves are perceived as an expression of inflammation, although a direct correlation between enlarged nerves and histopathological alteration has not yet been reported in the literature. In clinical routine, patients with axonal damage can still show disease activity, which could correspond to a recurring inflammation. However, since clear evidence is pending, it still can only be assumed that enlarged nerves in patients with CIDP reflect inflammation even in an advanced disease stage with axonal damage.

### Score Development

For developing a new score, the first step is to identify suitable test parameters. We had to find nerve sites, which fulfill the following requirements: First, easy and quick to measure, and second, each site should show significant differences in the values compared in our two groups.

We scrutinized nerve sites that were already tested in previous scores like BUS (18). Three mixed nerves (medial, ulnar, and radial) and a mainly sensory nerve (sural) were part of our examination protocol. We analyzed proximal and distal measurements, which are important because of the patchy multifocal CSA enlargements of patients with CIDP in previous studies (5). The sural nerve was found to be suitable for the identification of CIDP in previous studies, because of its sonomorphological enlargement due to the sensorimotor type of affection occurring in this type of immune-mediated polyneuropathy (19, 20). Furthermore, studies have shown that the sural (and the radial) nerve of patients with diabetes caused neuropathy did not show any significant CSA increase (21), which underlines its suitability regarding our research question. Therefore, we decided to include this measuring point. However, the patients with CIDP showed higher CSA values in each site, but no significant increase of CSA of the sural nerve. The possible explanation being a more difficult measurement due to the small diameter of the nerve and the associated inaccuracy.

In previous studies, acquired axonal neuropathies, such as in patients with diabetes showed higher CSA values of nerve sites, which are exposed to compression (21, 22). Therefore, we decided to exclude possible compression sites to ensure high test accuracy.

From these findings, we developed the aBUS and calculated the goodness-of-fit criteria for different cut-off values, and set a cut-off at ≥2 points, which is associated with a medium sensitivity but with high specificity.

### Use in Clinical Practice

The ultrasound examination is a practical, affordable, and effective method to detect morphological nerve alterations in different forms of peripheral neuropathies (15) and could be used as a complementary method to the NCS to allow evaluation of both function and morphology of investigated nerves (14). The advantages of the new score are similar to those of BUS: (a) easy administration, (b) economy of time (10–15 min), (c) high specificity, and (d) lack of side effects or pain for the patients (5). Possible anatomical limitations like obesity may produce difficulties in the measurement of reliable CSA values and thus also in the determination of the aBUS.

In contrast to our diagnostic pathway, Herraets et al. (8) recommended an application of nerve ultrasound as a screening test, followed by NCS to identify potentially treatment-responsive patients without sonographic abnormalities. The reason for the proposed approach is a high sensitivity of around 85% of their short ultrasound protocol (8). The different results of our studies might be due to the fact that we scrutinized patients with secondary axonal damage, whereas Herrats et al. (8) included patients with suspected symptoms at the beginning of the course of the disease.

In terms of subgroup analysis, we compared the sensitivity and specificity of the diagnostic pathway for typical CIDP and DADS. We showed that our findings can be applied not only for patients with tCIDP but also for patients with DADS (Table 5). This is significant because the clinical presentation of NIAP and DADS is particularly similar.

### Strengths and Limitations

The strength of our study is the detailed analysis of NCS and CSA measurements of a large cohort with typical CIDP and the atypical variant DADS with a control group consisting of many patients with the most prevalent NIAP. Another strength of the study is its placement in the diagnostic pathway to verify compatibility with everyday clinical practice.

A limitation of our ultrasound examination is that we did not analyze brachial plexus or cervical roots, whereas previous studies showed enlargement, especially in proximal nerve segments and roots (8, 22). Reliable examinations of these sites were difficult due to obesity, short neck, and a challenging standardization. This is paralleled by Herraets et al. (8) and Goedee et al. (22) who excluded the technically more challenging assessment of spinal roots, which previously showed high inter-observer variability and lower diagnostic yield.

Furthermore, Goedee et al. (22) considered treatment-naive patients, whereas we observed a cohort characterized by heterogeneity of disease duration and treatment regimes. Both aspects might influence the sonographic pattern of peripheral nerve enlargement, which makes our results valuable as they represent daily clinical life (12, 17, 23, 24). We did not analyze data of height, body weight, and age, as the effects of these demographic data on CSA are still under discussion (25–28).

In this study, we have disregarded aspects such as CSA variability, echogenicity, and fascicle size. Goedee et al. (22) found significantly higher echogenicity and larger fascicle size in large arm nerves of patients with CIDP than in those with axonal neuropathies. However, these characteristics rather have prognostic value and are used for disease and treatment monitoring (25).

Another limitation of our study is the retrospective design.

The NCS of the NIAP in our study was not performed as detailed as required for the diagnosis of CIDP. Nonetheless, regarding the EFNS/PNS criteria (2), it can thus be assumed that even more patients than indicated above fulfill the electrophysiological criteria. Another limitation of our study is that recently, a new revision of EFNS/PNS criteria was published (29). In these, nerve ultrasound was included for the first time as a diagnostic tool. However, sonographical characteristics of CIDP with secondary axonal damage are still not regarded in the criteria, and electrophysiological criteria were not changed essentially, which is why our study is still of interest.

## Conclusion

Nerve ultrasound is a helpful tool to differentiate between patients with CIDP with secondary axonal damage and NIAP. Diagnosis of CIDP such as typical and atypical CIDP, in which electrophysiological EFNS/PNS criteria result in only “possible” or “probable” diagnosis due to secondary axonal damage can be supported by nerve ultrasound.

It can facilitate the diagnosis of inflammatory neuropathy by using aBUS even in case of axonal damage. If EFNS/PNS criteria are fulfilled as possible or probable and aBUS is at least 2 points, an inflammatory genesis can be assumed, and appropriate therapy can be initiated.

## Data Availability Statement

The raw data supporting the conclusions of this article will be made available by the authors, without undue reservation.

## Ethics Statement

The studies involving human participants were reviewed and approved by Ethikkomitee der Ruhr-Universität Bochum. The patients/participants provided their written informed consent to participate in this study. Written informed consent was obtained from the individual(s) for the publication of any potentially identifiable images or data included in this article.

## Author Contributions

JB and JM contributed to data collection, statistical analysis, drafting, and revising the manuscript. JM supported with the basic idea. TG, HM, YB, and DA involved in data collection and revising the manuscript. AC revised the manuscript. M-SY and RG added critical comments during data collection, drafting, and manuscript revision. RG involved in technical infrastructure. KP and AF supported with the basic idea, critical comments during data collection, drafting, and manuscript revision. AF performed the statistical analysis. All authors contributed to the article and approved the submitted version.

## Conflict of Interest

JM has no personal pecuniary interests to disclose, received travel grants and supply from Biogen, Novartis, Celgene (BristolMyersSquibb), Teva and Eisai, and the author's research is funded by Klaus Tschira Foundation, Hertie Foundation and Ruhr-University, Bochum (FORUM-program), none related to this study. TG received travel reimbursement from Sanofi Genzyme and Biogen Idec, none related to this manuscript. M-SY has received speaker honoraria from CSL Behring and Grifols, a scientific grant from CSL Behring, none related to this manuscript. RG serves on scientific advisory boards for Teva Pharmaceutical Industries Ltd., Biogen Idec, Bayer Schering Pharma, and Novartis; has received speaker honoraria from Biogen Idec, Teva Pharmaceutical Industries Ltd., Bayer Schering Pharma, and Novartis; serves as an editor for Therapeutic Advances in Neurological Diseases and on the editorial boards of Experimental Neurology and the Journal of Neuroimmunology; and receives research support from Teva Pharmaceutical Industries Ltd., Biogen Idec, Bayer Schering Pharma, Genzyme, Merck Serono, and Novartis, none related to this manuscript. KP received travel funding and speaker honoraria from Biogen Idec, Novartis and Bayer Schering Pharma and funding from the Ruhr-University, Bochum (FORUM-program), none related to this study. AF received research funding from Georgius Agricola Stiftung Ruhr and Ruhr-University, Bochum (FORUM-program), received honoraria and travel grants from Novartis AG, Sanofi and Eisai GmbH, none related to this study, and owns shares of Fresenius SE & Co., Gilead Sciences, Medtronic PLC and Novartis AG. The remaining authors declare that the research was conducted in the absence of any commercial or financial relationships that could be construed as a potential conflict of interest.

## Publisher's Note

All claims expressed in this article are solely those of the authors and do not necessarily represent those of their affiliated organizations, or those of the publisher, the editors and the reviewers. Any product that may be evaluated in this article, or claim that may be made by its manufacturer, is not guaranteed or endorsed by the publisher.
